# Characteristics and Outcome of Elderly Patients (>55 Years) with Acute Lymphoblastic Leukemia

**DOI:** 10.3390/cancers14030565

**Published:** 2022-01-23

**Authors:** Daniela V. Wenge, Klaus Wethmar, Corinna A. Klar, Hedwig Kolve, Tim Sauer, Linus Angenendt, Georg Evers, Simon Call, Andrea Kerkhoff, Cyrus Khandanpour, Torsten Kessler, Rolf Mesters, Christoph Schliemann, Jan-Henrik Mikesch, Christian Reicherts, Monika Brüggemann, Wolfgang E. Berdel, Georg Lenz, Matthias Stelljes

**Affiliations:** 1 Department of Medicine A, Hematology, Oncology, Hemostaseology, Pneumology, University Hospital Muenster, 48149 Muenster, Germany; DanielaV_Wenge@dfci.harvard.edu (D.V.W.); Klaus.Wethmar@ukmuenster.de (K.W.); Corinna.Klar@mjh-greven.de (C.A.K.); Hedwig.Kolve@ukmuenster.de (H.K.); Linus.Angenendt@ukmuenster.de (L.A.); Georg.Evers@ukmuenster.de (G.E.); Simon.Call@ukmuenster.de (S.C.); Andrea.Kerkhoff@ukmuenster.de (A.K.); Cyrus.Khandanpour@ukmuenster.de (C.K.); Torsten.Kessler@ukmuenster.de (T.K.); Rolf.Mesters@ukmuenster.de (R.M.); Christoph.Schliemann@ukmuenster.de (C.S.); Jan-Henrik.Mikesch@ukmuenster.de (J.-H.M.); Christian.Reicherts@ukmuenster.de (C.R.); berdel@uni-muenster.de (W.E.B.); Georg.Lenz@ukmuenster.de (G.L.); 2 Dana-Farber Cancer Institute, Department of Pediatric Oncology, Harvard Medical School, Boston, MA 02215, USA; 3 Department of Medicine V, Hematology, Oncology, Rheumatology, University Hospital Heidelberg, 69120 Heidelberg, Germany; Tim.Sauer@med.uni-heidelberg.de; 4 Department of Biosystems Science and Engineering, ETH Zürich, 4058 Basel, Switzerland; 5 Department of Medicine II, Hematology and Oncology, University Hospital Schleswig Holstein, 24105 Kiel, Germany; m.brueggemann@med2.uni-kiel.de

**Keywords:** acute lymphoblastic leukemia, elderly patients, clinical data

## Abstract

**Simple Summary:**

Disease-specific mortality of acute lymphoblastic leukemia (ALL) increases with age. So far, only a few analyses have investigated disease characteristics of elderly patients (>55 years) with newly diagnosed ALL. The aim of our retrospective study was to evaluate the treatment results of 93 elderly patients who received intensive chemotherapy between May 2003 and October 2020. We identify poor performance status and older age at the time of diagnosis as risk factors for inferior outcomes, while ALL immunophenotype, BCR::ABL1 status, the complexity of karyotype, and intensity of treatment did not significantly affect overall survival (OS). With 17.3% of patients dying while in complete remission (CR), an event-free survival (EFS) and OS of 32.9% and 47.3% at 3 years, our data suggest that intensive treatment of elderly ALL patients is feasible but associated with significant toxicity. These results underline the need for novel, less toxic treatment approaches for this vulnerable cohort of patients.

**Abstract:**

Prognosis of elderly ALL patients remains dismal. Here, we retrospectively analyzed the course of 93 patients > 55 years with B-precursor (*n* = 88) or T-ALL (*n* = 5), who received age-adapted, pediatric-inspired chemotherapy regimens at our center between May 2003 and October 2020. The median age at diagnosis was 65.7 years, and surviving patients had a median follow-up of 3.7 years. CR after induction therapy was documented in 76.5%, while the rate of treatment-related death within 100 days was 6.4%. The OS of the entire cohort at 1 and 3 year(s) was 75.2% (95% CI: 66.4–84.0%) and 47.3% (95% CI: 36.8–57.7%), respectively, while the EFS at 1 and 3 years(s) was 59.0% (95% CI: 48.9–69.0%) and 32.9% (95% CI: 23.0–42.8%), respectively. At 3 years, the cumulative incidence (CI) of relapse was 48.3% (95% CI: 38.9–59.9%), and the CI rate of death in CR was 17.3% (95% CI: 10.9–27.5%). Older age and an ECOG > 2 represented risk factors for inferior OS, while BCR::ABL1 status, immunophenotype, and intensity of chemotherapy did not significantly affect OS. We conclude that intensive treatment is feasible in selected elderly ALL patients, but high rates of relapse and death in CR underline the need for novel therapeutic strategies.

## 1. Introduction

Although significant treatment advances in pediatric and young adult patients with acute lymphoblastic leukemia (ALL) have been achieved over the last decades, the outcome for elderly ALL patients (commonly defined as older than 55–60 years of age) remains dismal [1,2,3,4,5,6,7,8,9,10,11]. This is reflected by the observation that >50% of ALL-related deaths occur in patients > 55 years [1,4], while this group of patients accounts for only 20–30% of entire ALL cases [1,2,11]. So far, detailed data on disease characteristics, applied treatment regimens, and outcome of this group of patients is limited, as older patients participate in clinical trials less often [3,11,12] and prospective trials specifically designed to evaluate therapeutic regimens for elderly ALL patients are scarce [1,3,5]. The poor outcome of this group of patients most likely originates from both patient- and disease related characteristics [1,3,5].

Elderly ALL patients represent a heterogeneous group regarding performance status, medical history, and comorbidities and thus largely differ in treatment tolerability [13]. In many cases, patients’ frailty precludes administration of intensive chemotherapy, and therefore sufficient antileukemic activity cannot be attained [1,3,4,8,14]. Older age has been reported as a risk factor for the poorer outcome, even in the group of elderly patients considered suitable to receive intensive chemotherapy [11,15].

Compared to younger patients, elderly ALL patients present with specific features of ALL biology, including higher proportions of B-ALL immunophenotype, positivity for the BCR::ABL1 transcript, complex karyotype, TP53 mutations, and therapy-related disease [1,3,4,5,15,16,17,18]. In some analyzed cohorts, BCR::ABL1 positive ALL represents the largest disease subset in elderly ALL patients [4]. Interestingly, disease characteristics associated with high proliferation, such as hyperleukocytosis or large extramedullary involvement, seem to be less common in elderly ALL patients than in younger patients [3,5].

Due to the high rate of substantial comorbidities, previous reports estimated that less than 50% of elderly ALL patients received intensive chemotherapy with curative intent [11]. In Germany, pediatric-inspired intensive induction chemotherapy regimens, according to recommendations by the German multicenter ALL study group (GMALL), consist of corticosteroids, vincristine, an anthracycline, cytarabine, cyclophosphamide, and often methotrexate as well as asparaginase [3,11]. Treatment-related deaths mainly occur during induction therapy [4], and dose reductions, delay, or discontinuation of therapy are common in the group of elderly ALL patients [11]. Allogeneic hematopoietic stem cell transplantation (HSCT) has been reported as an important therapeutic option in the treatment of a subgroup of elderly ALL patients [19], but the selection of patients suitable for HSCT and the optimal post-remission strategy in this group of patients remain controversial [11,14,20].

Here we provide detailed data on disease characteristics, course of treatment, prevalence of risk factors, and outcome of a single-center cohort of 93 B-precursor or T-ALL patients > 55 years receiving age-adjusted intensive chemotherapy regimens.

## 2. Materials and Methods

We retrospectively analyzed data by reviewing patient records from 106 consecutive patients (pts) treated for precursor ALL at the University Hospital Muenster, Germany, between May 2003 and October 2020. The Charlson Comorbidity Index (CCI) [21] and the Eastern Cooperative Oncology Group (ECOG) performance status [21,22] at the time of diagnosis were calculated using information from the patients’ medical records. The retrospective analysis of patient-related data was approved by the local ethics committee (file number 2020-729-f-S).

The primary endpoints analyzed in this study were OS and EFS calculated from the day of the first diagnosis. EFS was defined as survival without relapse or progressive disease—the latter referring to patients with primary refractory ALL. For a subgroup analysis of patients receiving an allogeneic HSCT, the day of transplantation was defined as day 0 for calculation of OS and RFS after transplant. OS was defined as survival until death from any cause or time of final follow-up, as of 24 June 2021. RFS was defined as survival without relapse for patients who achieved a complete remission (CR; defined as <5% blasts in the bone marrow). Secondary endpoints were cumulative incidence of relapse (CIR) and non-relapse mortality (NRM). CIR was defined as time to leukemic recurrence after achieving complete remission. NRM was defined as death in continuous remission, i.e., death from any other cause than relapse or refractory disease and without the occurrence of a previous relapse. The term NRM, when used with a percentage, referred to the number of non-relapse deaths over the number of patients in the sample evaluated. OS, EFS, and RFS were analyzed using the Kaplan-Meier method. Cumulative incidence curves were used to estimate CIR and NRM. Patients with primary refractory disease were excluded from the calculation of cumulative incidences. Results are shown, including the 95% confidence interval.

Univariate analyses were performed using the log-rank-test for EFS and OS, Cox proportional hazards model for OS, and Gray’s test for cumulative incidences. For descriptive statistics, Pearson’s Chi-squared test for categorical and Wilcoxon’s rank sum test for continuous variables were utilized. Variables with a *p* < 0.2 in univariate analysis were included in multivariate analyses (metric variables utilized as continuous parameters), performed using the Cox proportional hazards model. Statistical analyses were performed with the IBM SPSS statistics version 27 (Armonk, NY, USA) and XLSTAT 2016 (New York, NY, USA).

## 3. Results

### 3.1. Patient Characteristics

**Figure 1:**
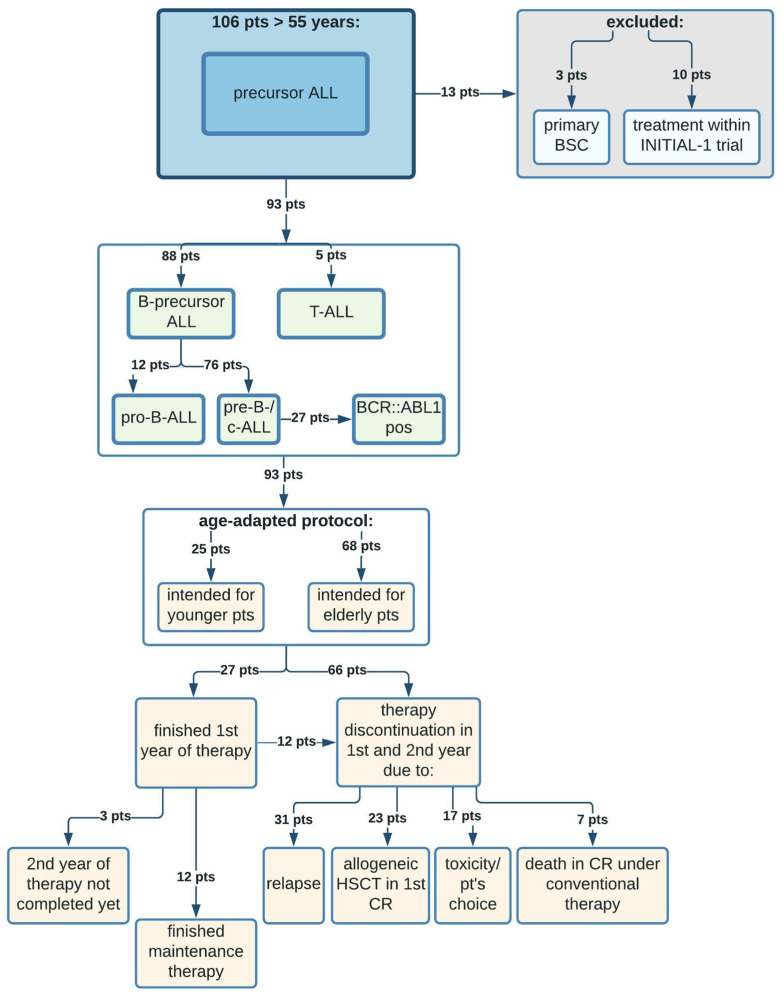
Consort diagram of all patients > 55 years treated for ALL at the University Hospital Muenster, Germany. BSC = best supportive care, pts = patients.

ALL patients treated within the INITIAL-1 study (NCT03460522, [23]; 10 pts) and patients who primarily received best supportive care (BSC) (3 pts) were excluded from further analysis (Figure 1).

Among the remaining 93 intensively treated patients aged >55 years with precursor ALL, 88 patients were diagnosed with B-ALL and 5 patients with T-ALL (Figure 1). Seventy-six patients presented with a pre-B- or common-ALL (c-ALL), of which 27 patients were BCR::ABL1 positive and 12 patients presented with a pro-B-ALL.

Out of the 93 patients, eighty-four (87.5%) were admitted to our center to receive first-line induction chemotherapy. Of these, eight patients (8.3%) were referred for salvage treatment and four patients (4.1%) for allogeneic HSCT. Patients were treated according to the respectively valid recommendations of the GMALL study group.

The median patient age at diagnosis was 65.7 years (ranging from 55.2–85.1 years) (Table 1). 45.8% of all patients presented with an ECOG status of 0–1, 49.4% had an ECOG status of 2, and 4.8% of the patients had an ECOG status of 3. The CCI was 0 in 42.4% of the patients, 1 (43.4% of the patients), or ≥2 (29.4% of the patients) (Table 1).

Of all patients, 10.7% presented with therapy-related ALL after cytotoxic treatment of a primary malignancy (4 pts with prior breast cancer). In addition, 3 patients had prior malignancy ALL (pm-ALL) treated with surgery only.

26.7% of the patients had one or more risk factors such as hyperleukocytosis or pro-B-ALL phenotype (Table 1). 14.1% of all patients presented with extramedullary ALL manifestations and 7.3% with central nervous system (CNS) infiltration (Table 1).

When comparing patients with BCR::ABL1 positive and BCR::ABL1 negative B-precursor ALL for several disease characteristics, including age, sex, ECOG, CCI, blood levels at diagnosis, karyotype, extramedullary manifestations, CNS infiltration, risk factors, and the rate of therapy-related ALL, we observed significantly higher leukocyte counts at diagnosis in the subgroup of BCR::ABL1 positive patients (*p =* 0.005) (Table 1). Apart from that, no other significant differences could be detected.

The median follow-up of all patients was 2.2 years (range 24 days–16.9 years), and the median follow-up of surviving patients was 3.7 years (range 8.8 months–16.9 years), respectively.

### 3.2. Intensive Treatment with Conventional Chemotherapy

For intensive chemotherapy, age-adapted pediatric-inspired treatment regimens, according to the recommendations of the GMALL for younger (18–55 years, 25 pts) or elderly patients (>55 years, 68 pts), were applied (Figure 1 and Table 2). Patients treated according to the recommendations for younger patients had a median age of 59.4 years (range 55.2–74.7 years), and those treated according to the recommendations for elderly patients had a median age of 68.8 years (range 56.4–85.1 years).

In general, the treatment regimens consisted of two cycles of induction therapy followed by consolidation, reinduction, and consolidation therapy cycles in the 1st year and consecutive maintenance therapy in the 2nd year of treatment. The induction regimen and the reinduction cycle administered after the 2nd cycle of consolidation therapy included dexamethasone, cyclophosphamide, vincristine, idarubicin, and cytarabine combined with intrathecal (i.th.) CNS prophylaxis (dexamethasone, cytarabine and methotrexate). Consolidation consisted of alternating cycles of high-dose methotrexate/asparaginase and high-dose cytarabine, as well as i.th. CNS prophylaxis. Additionally, rituximab was administered depending on the treatment regimen: to patients with BCR::ABL1 negative B-precursor ALL and >20% CD20+ blasts (if treated according to the recommendations for elderly patients) as well as to all BCR::ABL1 negative and those BCR::ABL1 positive patients with >20% CD20+ blasts. Nonetheless, these recommendations were not consistently followed throughout the study period, as rituximab was also frequently administered to patients with <20% CD20+ blasts.

All 27 patients with a BCR::ABL1 transcript received first-line treatment with the tyrosine kinase inhibitor (TKI) imatinib, either in addition to conventional induction chemotherapy (according to the recommendations for younger ALL patients) or as monotherapy in induction therapy (according to the recommendations for elderly ALL patients) (Table 2). Remission controls (including minimal residual disease (MRD) status and BCR::ABL1 transcript levels for patients that had tested positive at initial diagnosis) were performed before all treatment cycles, after last consolidation, and every three months during maintenance therapy. Fifteen patients discontinued imatinib treatment and switched to dasatinib as a second-line treatment—10 patients due to lack of response and 5 patients because of drug intolerance (nausea, vomiting, and diarrhea) (Table 2).

Of all patients in our cohort, 89.7% received i.th. therapy, 24.4% underwent prophylactic cranial irradiation, and 4.4% received involved site irradiation (Table 2).

The CR rate after induction therapy was 76.5% among those patients receiving their initial induction therapy at our center (Table 2). MRD status was analyzed by a quantitative real-time PCR in 44 of these patients, and 19 patients had an MRD negative CR after induction therapy. The CR rate after induction therapy was highest in the group of BCR::ABL1 positive patients (81.8%).

The rate of early death after intensive therapy (within 100 days after the start of treatment) was 6.4%, and 24.7% of the patients died within one year after diagnosis (Table 2). The 3 patients, who did not receive intensive chemotherapy due to poor performance status or patients’ preference, died within 2 months (20, 51, and 55 days after diagnosis). We found that 27 of 93 patients (29.0%) finished the first year of treatment (Figure 1). Subsequent maintenance therapy was administered to 12 patients (12.9%). The reasons for discontinuation of conventional treatment in the first and second year were relapsed disease (31 pts), allogeneic HSCT in 1st CR (23 pts), toxicity/patients’ preference (17 pts), and death in CR (7 pts, patients receiving an allogeneic HSCT excluded). At the time of data analysis, 3 patients had not yet completed the second year of therapy (Figure 1).

In both BCR::ABL1 positive and BCR::ABL1 negative patients, no significant differences in outcome could be detected for patients receiving more or less intensive chemotherapy (i.e., receiving protocols intended for younger vs. elderly patients), application of i.th. therapy, or irradiation (Table 2).

### 3.3. Survival Outcomes

**Figure 2:**
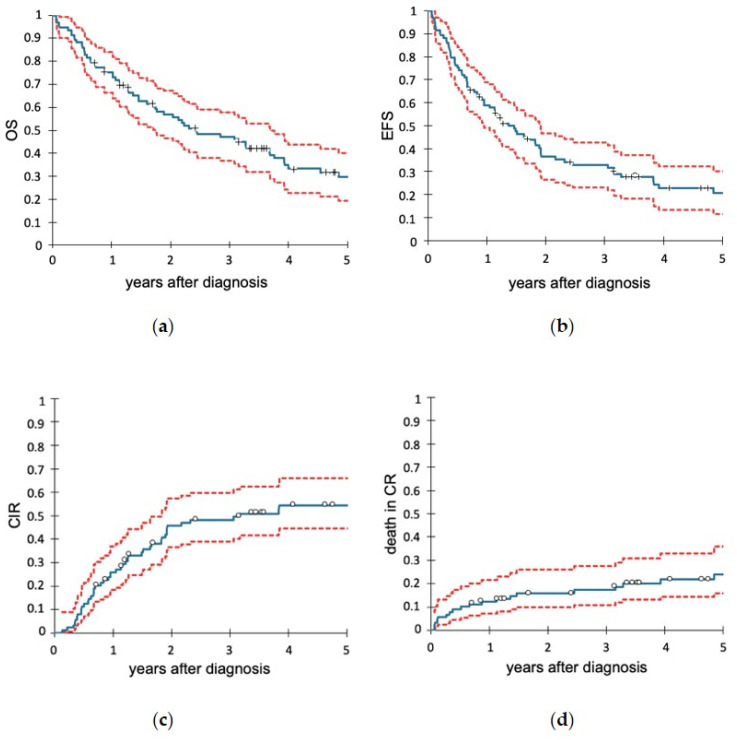
Outcomes of all intensively treated patients with B-precursor or T-ALL (*n* = 93). (**a**) Overall survival (OS). (**b**) Relapse-free survival (RFS). (**c**) Cumulative incidence of relapse (CIR). (**d**) Death in complete remission (NRM). In the diagrams, the blue lines show the Kaplan-Meier curves, and the red lines indicate the respective 95% confidence intervals.

OS of the entire cohort at 1 and 3 year(s) was 75.2% (95% CI: 66.4–84.0%) and 47.3% (95% CI: 36.8–57.7%), respectively (Figure 2a). EFS at 1 and 3 year(s) was 59.0% (95% CI: 48.9–69.0%) and 32.9% (95% CI: 23.0–42.8%), respectively (Figure 2b).

The cumulative incidence of relapse at 1 and 3 year(s) was 26.0% (95% CI: 18.4–36.8%) and 48.3% (95% CI: 38.9–59.9%), respectively (Figure 2c), with a median time to relapse of 11.4 months (range 1.3–46.7 months). The cumulative incidence of death in CR (NRM) at 1 and 3 year(s) was 12.4% (95% CI: 7.1–21.5%) and 17.3% (95% CI: 10.9–27.5%), respectively (Figure 2d). The main causes of death in CR among 20 of the 25 patients with data available were treatment toxicity (25.0%) and infections (50.0%).

Of note, 14 of the 45 patients with relapsed B-precursor ALL received salvage treatment, including blinatumomab and/or inotuzumab ozogamicin. The most frequent salvage treatment applied other than blinatumomab and/or inotuzumab ozogamicin was fludarabine/cytarabine/idarubicine (12 pts), and 7 of the patients receiving blinatumomab and/or inotuzumab ozogamicin salvage therapy subsequently underwent allogeneic HSCT.

**Figure 3:**
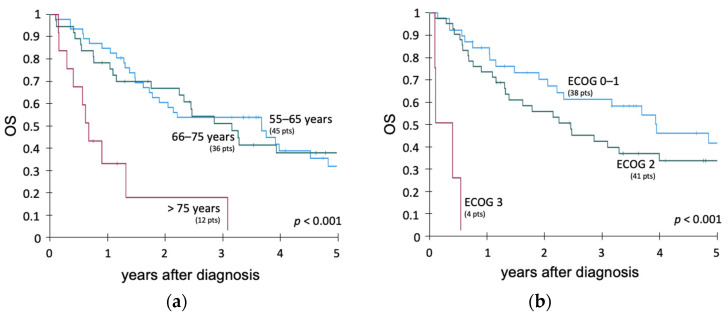
Overall survival of different subgroups of the entire patient cohort of 93 intensively treated patients (88 patients with B-precursor and 5 patients with T-ALL). (**a**) 5-year OS comparing age groups (45 pts 55–65 years, 36 pts 66–75 years, 12 pts > 75 years). (**b**) OS comparing ECOG (38 pts ECOG 0–1, 41 pts ECOG 2, 4 pts ECOG 3; data available for 83 pts).

For the entire patient cohort, age > 75 years (15 pts) was significantly associated with inferior OS (*p* < 0.001), whereas patients aged 55–65 years and 66–75 years had comparable outcomes (Figure 3a). An ECOG > 2 at initial diagnosis was significantly associated with shorter OS compared to an ECOG status of 0–1 vs. 2 (*p* < 0.0001, 4 pts vs. 38 pts vs. 41 pts). Comparing an ECOG of 0–1 vs. 2 showed no significant differences regarding OS (Figure 3b). Refractory disease after completion of induction therapy and first consolidation therapy was associated with a lower OS (*p* < 0.05). BCR::ABL1 status, ALL phenotype (T- or B-ALL), the intensity of conventional treatment applied (protocol originally intended for patients ≤ 55 years vs. >55 years), cytotoxic treatment for a previous malignancy or CCI (0 vs. 1 vs. 2–5) had no significant impact on OS and EFS. A comparison of OS among patients diagnosed with ALL during the first half of the study period (2003–2011, 47 pts) with those patients receiving their ALL diagnosis during the second half of the study period (2012–2020, 46 pts) showed no significant differences. 

In the univariate Cox proportional hazards model, an ECOG status > 2 and older age were associated with lower OS (*p* < 0.05) (Table 3). In a multivariate analysis, including variables with a *p* < 0.2 in univariate analysis, an ECOG status > 2 and older age remained significantly associated with inferior OS (*p* < 0.05) (Table 3).

### 3.4. Subgroup Analysis of the Patients Receiving an Allogeneic HSCT

A subgroup of 33 patients (34.4%) received an allogeneic HSCT as part of their ALL treatment (Table 4). The median age at the time of the allogeneic HSCT was 61.7 years. With 59.3%, the rate of allogeneic HSCT was highest in the subgroup of patients who presented with a BCR::ABL1 transcript at initial diagnosis (Table 2).

In total, 23 patients (69.7%) were transplanted in 1st CR, 7 patients in 2nd CR (21.2%), and 3 patients (9.1%) were transplanted with active disease (Table 4). A total of 53.5% of the patients transplanted in CR were MRD negative. A total of 31 patients received a conditioning therapy with fludarabine and 8 Gy fractioned total body irradiation. Most patients were transplanted from matched unrelated donors (90.9%). Acute and chronic graft-versus-host disease (GvHD) of any grade occurred in 48.5% and 33.3% of the patients, respectively, while severe acute (>grade 2) and severe chronic GvHD occurred in 27.3% and 24.2% of the patients, respectively.

**Figure 4:**
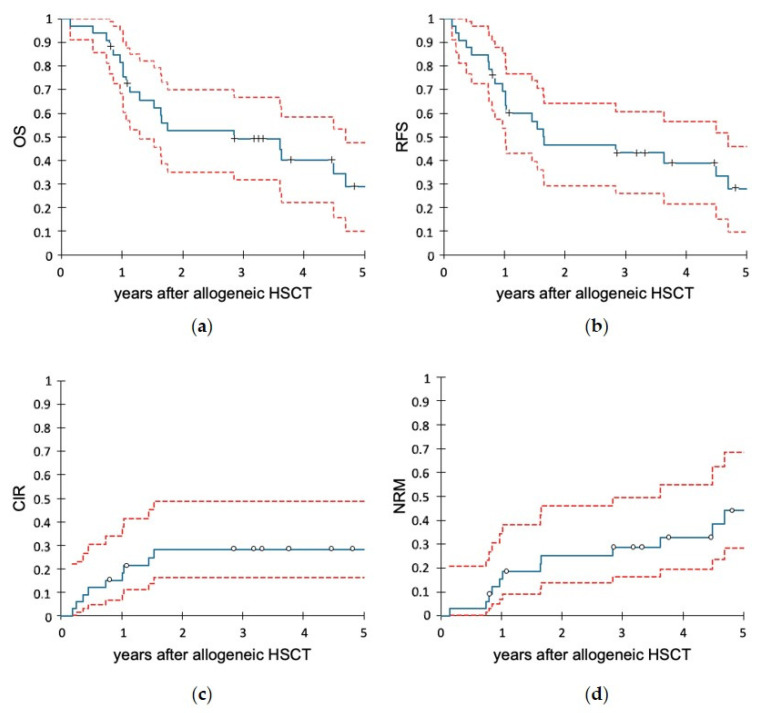
Outcomes of all patients receiving an allogeneic HSCT (33 pts). Follow-up starts with the time of allogeneic HSCT. (**a**) Overall survival (OS). (**b**) Relapse-free survival (RFS). (**c**) Cumulative incidence of relapse (CIR). (**d**) Non-relapse mortality (NRM). In the diagrams, the blue lines show the Kaplan-Meier curves, and the red lines indicate the respective 95% confidence intervals.

OS of the subgroup of patients receiving an allogeneic HSCT at 1 and 3 year(s) after allogeneic HSCT was 81.6% (95% CI: 68.3–94.9%) and 49.2% (95% CI: 31.7–66.8%), respectively (Figure 4a). RFS at 1 and 3 year(s) was 69.4% (95% CI: 53.6–85.2%) and 43.3% (25.9–60.4%) respectively (Figure 4b). 

The cumulative incidence of relapse after allogeneic HSCT at 1 and 3 year(s) was 15.2% (95% CI: 6.8–33.9%) and 28.1% (95% CI: 16.2–48.9%), respectively (Figure 4c). The cumulative incidence of NRM at 1 and 3 year(s) was 15.4% (95% CI: 6.9–34.5%) and 28.6% (95% CI: 16.4–49.7%), respectively (Figure 4d).

## 4. Discussion

Here, we provide a detailed analysis of disease characteristics and outcomes for 93 elderly patients (>55 years) with B-precursor or T-ALL diagnosed between 2003 and 2020.

We assume that the cohort treated in our center clearly overrepresents fit elderly patients considered suitable for intensive chemotherapy by external physicians, while unfit patients may not have been referred. Only 3.1% of all patients admitted to our center were considered unfit for intensive chemotherapy and hence primarily received the best supportive care.

This assumption is supported by a recent population-based and thus unselected study cohort of ALL patients > 55 years from Sweden. A total of 80% of the patients in this cohort received intensive treatment and 20% followed a palliative approach [24], which is comparable to the rates of intensive and palliative therapy reported in the retrospective Mayo Clinic study of ALL patients > 60 years [2]. An analysis of Medicare data among patients ≥ 66 years reported that only 41.2% of the patients received intensive chemotherapy [25]. Furthermore, early death rates of 20–40% have been reported for elderly ALL patients [11], which are clearly higher than the early death rate of 6.4% observed in our cohort.

Patients with t-ALL seem to be slightly over-represented in our study as compared to the data available so far that report incidences of 2.5–9% [26,27,28]. This observation might be partly explained by the fact that previous reports refer to all adult patients and do not specifically focus on the elderly. Moreover, patients with t-ALL have been previously shown to be older than patients with de novo ALL [28].

In the group of intensively treated patients, poor performance status and older age at diagnosis were significantly associated with shorter OS in our analysis. They should be considered as risk factors for elderly ALL patients. These findings align with the Swedish study that reported age > 75 years at diagnosis as a risk factor for poorer outcomes [24]. The Mayo Clinic study similarly identified an ECOG status ≥ 2, but also a high white blood cell count and a high LDH as risk factors [2]. Older age has previously been recognized as an independent risk factor [5]. The GMALL elderly 01/2003 trial, the largest prospective trial specifically designed for elderly ALL patients so far (268 pts included), reported age, comorbidity score, and ECOG status as risk factors for shorter OS [29].

Interestingly, BCR::ABL1 status had no impact on prognoses in our patient cohort, which is in line with previous studies showing that the introduction of TKIs eliminates the negative impact of BCR::ABL1 positivity on prognosis [24,30]. Historically, the presence of the Philadelphia chromosome represented a risk factor, but recent studies found no influence of BCR::ABL1 status on outcome [24,31,32] or even associated the BCR::ABL1 transcript with a favorable prognosis [2,33]. In addition to the BCR::ABL1 status, the MRD level after the completion of induction therapy and after first consolidation chemotherapy now seems to be the most significant factor determining treatment outcome [3].

The OS rates of 75.2% at 1 year and of 47.3% at 3 years, as well as the CR rate of 76.5% among intensively treated patients in our cohort, are in line with the results of the few previous prospective studies specifically designed for elderly ALL patients. These were completed between 2007 and 2017 and showed OS rates of 24–66% at 2 years and 23% at 5 years [1]. CR rates at any time were 58–90% [1]. The two largest prospective studies, including elderly ALL patients (MD Anderson experience with hyper-CVAD [34]) and the MRC UKALL XII/ECOG2993 trial [15]), reported a 5-year OS of about 20%, which appears to be somewhat lower than the OS rate observed in our cohort. Comparability might be limited, among other factors, by the different therapeutic regimens used and the availability and standard of supportive care at the time of treatment. The most recently available reports—such as an analysis of the SEER (surveillance, epidemiology, and end results) database in the US [7] and the above mentioned Swedish population-based study of 115 elderly ALL patients [24]—suggest that real-world outcomes may be worse than reported in analyses originating from tertiary care centers such as ours [1].

In our analysis, the type of treatment regimen applied (i.e., originally intended for patients ≤ 55 years or >55 years) did not significantly affect the outcome. Thus, it remains an open question whether less intensive treatments may be sufficient to treat patients > 55 years. Current recommendations of the GMALL propose the “elderly protocol” for all patients > 55 years—even if biologically judged younger. Interestingly, the Swedish population-based study, including patients diagnosed with ALL between 2005 and 2012, neither showed the clinical benefit of a newly introduced age-adapted protocol for older patients [24].

Similar to the lack of consensus regarding the conventional therapeutic regimen, criteria for selecting patients suitable for allogeneic HSCT and the optimal conditioning therapy have not been defined [3]. Assessment tools to evaluate candidates for allogeneic HSCT, in general, have been developed, with some scores specifically designed for elderly ALL patients [1,35,36]. Current data suggest that reduced-intensity conditioning should be used for elderly ALL patients to reduce transplant-related mortality without apparent reduction of clinical benefit [30,37]. Outcomes in our patient cohort appeared to be somewhat better than those reported in the retrospective studies of the Swedish registry, the Mayo Clinic, and the European Group for Blood and Marrow Transplantation [2,24,38].

As poor tolerance of intensive chemotherapy regimens due to toxicity is a common problem in elderly ALL patients [3,4,11], low-intensity or “chemotherapy-free” regimens may represent efficient therapeutic strategies to reduce extensive toxicity [1]. Notably, the mere reduction of the chemotherapy by applying very attenuated doses resulted in rather low CR rates of about 50% in elderly ALL patients [39]. These results underline the need for novel approaches combining targeted and/or immune-based therapies to reduce toxicity while maintaining high antileukemic activity.

Novel agents like blinatumomab and inotuzumab ozogamicin are currently being tested in clinical trials and represent promising future therapeutic options, both as frontline and salvage therapy of B-cell precursor ALL patients [40]. Importantly, both antibodies demonstrated clinical benefit irrespective of patient age with an acceptable safety profile [4,41,42,43].

Due to small patient numbers in the respective subsets and heterogeneity of the treatment applied, no definite conclusions with regards to the impact of therapeutic options that became available during the study period—such as blinatumomab and inotuzumab ozogamicin as well as rituximab—on patients’ outcome can be drawn from our analysis. Regarding the entire patient cohort, outcomes seem to have improved only modestly between 2003 and 2020 since no significant changes in OS could be detected in those elderly ALL patients diagnosed in the first vs. the second half of the study period.

Promising clinical phase II trials for patients > 55 years with B-precursor ALL such as the INITIAL-1 trial of the GMALL study group evaluating the clinical efficacy and safety of replacing conventional first-line induction chemotherapy by inotuzumab ozogamicin [23,44] are currently ongoing. The INITIAL-1 trial showed promising treatment feasibility, remission rates, and outcome [23,44]. Similarly, the EWALL-BOLD trial evaluates replacing the second cycle of intensive induction chemotherapy with blinatumomab followed by sequential chemotherapy and further blinatumomab cycles to treat elderly ALL patients [45]. Apart from that, inotuzumab ozogamicin and blinatumomab combined with low-intensity chemotherapy have shown promising results in two phase II studies in the same patient group [46,47,48].

In the subgroup of BCR::ABL1 positive elderly ALL patients (>55 years), the combination of dasatinib with blinatumomab as a “chemotherapy-free” induction and consolidation regimen has recently been evaluated in the GIMEMA LAL 2116 D-ALBA trial [49]. The study was not specifically designed for elderly patients, but a relevant proportion accounted for this patient subgroup [49]. Treatment efficacy was promising with high CR and molecular response rates as well as low toxicity [49].

Consequently, the development of new treatment regimens for elderly ALL patients should focus on reducing chemotherapy intensity, especially during induction therapy, as treatment-related mortality is highest in this early treatment phase [5]. In addition to TKIs, which have replaced conventional first-line induction chemotherapy for BCR::ABL1 positive patients [18], bispecific or drug-conjugated antibodies could improve outcomes in the future in the same way.

## 5. Conclusions

Our retrospective analysis of elderly ALL patients (>55 years) shows that intensive treatment with conventional chemotherapeutic agents is feasible in patients of this subgroup. Nonetheless, high relapse rates and impaired survival underline the need for novel therapeutic strategies. Targeted therapies such as TKIs, bispecific antibodies, and antibody-drug conjugates may represent promising treatment options, even for frail and elderly patients.

## Figures and Tables

**Table 1:** Disease characteristics of the cohort of intensively treated elderly ALL patients (>55 years at diagnosis).

Disease Characteristics	All Patients *n* = 93	B-Precursor ALL			T-ALL *n* = 5
		All Patients *n* = 88	BCR::ABL1 pos. *n* = 27	BCR::ABL1 neg. *n* = 61	
**ALL subtype**, *n* ^(1)^ (%):					
pro-B-ALL	12/93 (12.9%)	12/88 (13.6%)	-	12/61 (19.7%)	-
c-ALL	69/93 (74.2%)	69/88 (78.4%)	27/27 (100.0%)	42/61 (68.9%)	-
pre-B-ALL	7/93 (7.5%)	7/88 (8.0%)	-	7/61 (11.4%)	-
T-ALL	5/93 (5.4%)	-	-	-	5/5 (100.0%)
**BCR::ABL1**, *n* (%):					
positive	27/93 (29.0%)	27/88 (30.7%)	27/27 (100.0%)	-	-
**Gender**, *n* (%):					
male	43/93 (46.2%)	41/88 (46.6%)	15/27 (55.6%)	26/61 (42.6%)	2/5 (40.0%)
female	50/93 (53.8%)	47/88 (53.4%)	12/27 (44.4%)	35/61 (57.4%)	3/5 (60.0%)
**Age at diagnosis** (years), median (range) (*n*):	65.7 (55.2–85.1) (93)	65.6 (55.2–85.1) (88)	63.4 (55.8–79.7) (27)	66.3 (55.2–85.1) (61)	70.2 (57.3–76.8) (5)
**ECOG at diagnosis**, *n* ^(1)^ (%):					
0	2/83 (2.4%)	2/78 (2.6%)	-	2/53 (3.8%)	-
1	36/83 (43.4%)	33/78 (42.3%)	11/25 (44.0%)	22/53 (41.5%)	3/5 (60.0%)
2	41/83 (49.4%)	40/78 (51.3%)	12/25 (48.0%)	28/53 (52.8%)	1/5 (20.0%)
3	4/83 (4.8%)	3/78 (3.8%)	2/25 (8.0%)	1/53 (1.9%)	1/5 (20.0%)
**CCI at diagnosis**, *n* (%):					
0	39/92 (42.4%)	36/87 (41.4%)	9/27 (33.3%)	27/60 (45.0%)	3/5 (60.0%)
1	26/92 (28.3%)	24/87 (27.6%)	9/27 (33.3%)	15/60 (25.0%)	2/5 (40.0%)
2	19/92 (20.7%)	19/87 (21.8%)	6/27 (22.2%)	13/60 (21.7%)	-
≥3	8/92 (8.7%)	8/87 (9.2%)	3/27 (11.1%)	5/60 (8.33%)	-
**Therapy-related ALL**, *n* (%):	10/92 (10.7%)	10/88 (11.4%)	4/27 (14.8%)	6/61 (9.8%)	-
**High-risk ALL**^(2)^, *n* (%):	65/90 (72.2%)	60/85 (70.6%)	27/27 (100.0%)	33/58 (56.9%)	5/5 (100.0%)
**Leukocytes at diagnosis** (1/nL), median (range) (*n*):	8.4 (0.8–713.0) (86)	8.2 (0.8–713.0) (81)	31.7 (2.3–713.0) (25)	5.6 (0.8–117.0) (56)	28.2 (1.7–40.4) (5)
**Hemoglobin at diagnosis** (g/dL), median (range) (*n*):	9.8 (4.7–15.7) (86)	9.6 (4.7–13.8) (81)	9.8 (4.7–14.0) (25)	9.6 (6.5–13.8) (56)	12.3 (9.8–15.7) (5)
**Thrombocytes at diagnosis** (1/nL), median (range) (*n*):	51 (6–618) (86)	50 (6–618) (81)	44 (7–618) (25)	53 (6–499) (56)	66 (11–207) (5)
**LDH at diagnosis** (U/L),	592 (135–6254)	579 (135–6254)	572 (135–3680)	675 (149–6254)	675 (252–6020)
median (range) (*n*):	(85)	(81)	(25)	(55)	(5)
**Bone marrow blasts at diagnosis** (%), median (range) (*n*):	86 (20–100) (78)	87 (20–100) (75)	85 (20–100) (23)	88 (25–96) (52)	80 (80–93) (3)
**Peripheral blasts at diagnosis** (%), median (range) (*n*):	36 (0–99) (73)	38 (0–99) (68)	52 (0–98) (23)	26 (0–99) (45)	4 (0–88) (5)
**Karyotype**, *n*/pts tested (%):					
normal	22/80 (27.5%)	20/77 (22.0%)	3/22 (13.6%)	17/55 (30.9%)	2/3 (66.6%)
1 or 2 aberrations	27/80 (33.8%)	26/77 (28.6%)	12/22 (54.5%)	14/55 (25.4%)	1/3 (33.3%)
complex (≥3 aberrations)	31/80 (38.7%)	31/77 (31.9%)	7/22 (31.8%)	24/55 (43.6%)	-
**Extramedullary ALL at diagnosis**, *n* (%):	13/92 (14.1%)	10/87 (11.5%)	1/26 (3.8%)	9/61 (14.8%)	3/5 (60.0%)
**CNS involvement at diagnosis**, *n* (%):	6/82 (7.3%)	6/78 (7.7%)	1/24 (4.2%)	5/54 (9.3%)	-

^(1)^ General remark: “*n*” indicates the number of patients with data available for the respective category. ^(2)^ ≥1 of the following disease characteristics: leukocytosis > 30.000/µL at diagnosis, t (9;22) or t (4;11), pro-B-ALL, MRD positivity after 1st consolidation, late CR (after >3 weeks, i.e., later than after 1st induction chemotherapy).

**Table 2:** Treatment characteristics of all elderly ALL patients (>55 years at diagnosis) with B-precursor and T-ALL receiving intensive chemotherapy (93 pts).

Treatment Characteristics	All Patients *n* = 93	B-Precursor ALL			T-ALL *n* = 5
		All Patients *n* = 88	BCR::ABL1 pos. *n* = 27	BCR::ABL1 neg. *n* = 61	
**Therapy regimen**, *n* ^(1)^ (%):					
intended for elderly patients	68/93 (73.1%)	64/88 (72.3%)	20/27 (74.1%)	44/61 (72.1%)	4/5 (80.0%)
intended for younger patients	25/93 (26.9%)	24/88 (27.3%)	7/27 (25.9%)	17/61 (27.9%)	1/5 (20.0%)
**i.th. therapy**, *n* (%):	78/87 (89.7%)	75/82 (91.5%)	24/25 (96.0%)	51/57 (89.5%)	3/5 (60.0%)
**Radiotherapy**, *n* (%):					
-prophylactic cranial irradiation	22/90 (24.4%)	22/85 (25.9%)	4/26 (15.3%)	18/59 (30.5%)	-
-other site of prim. RTx ^(2)^	3/90 (3.3%)	2/85 (2.4%)	-	2/59 (3.4%)	1/5 (20.0%)
-cranial irradiation for prim. CNS involvement	1/90 (1.1%)	1/85 (1.2%)	-	1/59 (1.7%)	-
**TKI**, *n* (%): -imatinib 1st line -dasatinib 2nd line -nilotinib and/or ponatinib 3rd/4th line ^(4)^	28/93 (30.1%) 15/93 (5.4%) 4/93 (4.3%)	28/88 (31.8%) 15/88 (17.0%) 4/88 (4.5%)	27/27 (100%) 15/27 (55.6%) 4/27 (14.8%)	1/61 (1.6%) ^(3)^ - -	0/5 (0%) - -
**Allogeneic HSCT**, *n* (%):	33/93 (35.5%)	32/88 (36.4%)	16/27 (59.3%)	16/61 (26.2%)	1/5 (20.0%)
**Remission status after induction**^(5)^, *n*/ pts tested (%):					
CR	62/81 (76.5%)	59/76 (77.6%)	18/22 (81.8%)	41/54 (75.9%)	3/5 (60.0%)
refractory	11/81 (13.6%)	10/76 (13.2%)	3/22 (13.6%)	7/54 (13.0%)	1/5 (20.0%)
not evaluated ^(6)^	8/81 (9.9%)	7/76 (9.2%)	1/22 (4.5%)	6/54 (11.1%)	1/5 (20.0%)
**MRD status after induction**^(7)^, *n*/ pts tested (%):					
positive	24/43 (55.8%)	23/42 (54.8%)	12/17 (70.6%)	11/25 (44.0%)	1/1 (100.0%)
negative	19/43 (44.2%)	19/42 (45.2%)	5/17 (29.4%)	14/25 (56.0%)	-
**Remission status after 1st consolidation**^(8)^, *n*/ pts tested (%):					
CR	52/71 (73.2%)	49/67 (73.2%)	17/22 (77.3%)	32/45 (71.1%)	3/4 (75.0%)
refractory	5/71 (7.0%)	4/67 (5.8%)	1/22 (4.5%)	3/45 (6.7%)	1/4 (25.0%)
not evaluated	14/71 (19.7%)	14/67 (21.0%)	4/22 (18.2%)	10/45 (22.2%)	-
**MRD status after 1st consolidation**^(9)^, *n*/pts tested (%):					
positive	19/37 (51.4%)	18/36 (50.0%)	12/16 (75.0%)	6/20 (30.0%)	1/1 (100.0%)
negative	18/37 (48.6%)	18/36 (50.0%)	4/16 (25.0%)	14/20 (70.0%)	-
**Primary refractory disease**, *n* (%):	4/93 (4.3%)	3/88 (3.4%)	1/27 (3.7%)	2/61 (3.3%)	1/5 (20.0%)
**Death**, *n* (%):					
<100 days after diagnosis	6/93 (6.4%)	5/88 (5.7%)	-	5/61 (8.2%)	1/5 (20.0%)
>100 days and ≤1 year after diagnosis	23/93 (24.7%)	21/88 (23.9%)	5/27 (18.5%)	16/61 (26.2%)	2/5 (40.0%)

i.th. = intrathecal, TBI = total body irradiation, RTx = radiotherapy, CNS = central nervous system, prim. = primary, discont. = discontinuation, TKI = tyrosine kinase inhibitor, CTx = chemotherapy, BSC = best supportive care, CR = complete remission, MRD = minimal residual disease, incl. = including. ^(1)^ General remark: “*n*” indicates the number of patients with data available for the respective category. ^(2)^ Primary RTx (before relapse/due to persisting disease manifestations): 1 patient, respectively, received irradiation of: contralateral testis/paraaortal/iliacal, intraspinal ALL manifestation, neuro-axis. ^(3)^ In this patient, an ETV6-PDGFRβ transcript was detected. ^(4)^ Among these, 1 patient received ponatinib due to the development of a BCR::ABL1 T315I mutation. ^(5)^ Concerning only the 81 patients with B-precursor and T-ALL receiving the induction therapy at the University Hospital Muenster, Germany, excluding 12 pts, who had been admitted only for salvage therapy or allogeneic HSCT. ^(6)^ Due to treatment discontinuation and/or death. ^(7)^ Included here: 62 patients treated at the University Hospital Muenster, Germany, with documented CR after 1st induction. ^(8)^ Concerning only the 71 patients receiving consolidation therapy at the University Hospital Muenster, Germany. ^(9)^ Included here: 52 patients treated at the University Hospital Muenster, Germany, with documented CR after 1st consolidation.

**Table 3:** Univariate and multivariate analysis of predictors of survival for 93 patients with B-precursor ALL or T-ALL receiving intensive treatment ^(1)^.

Predictors	*n*	Univariate Analysis		Multivariate Analysis	
		HR (95% CI)	*p*-Value	HR (95% CI)	*p*-Value
**B-precursor ALL/T-ALL**	88/5	1.10 (0.34–3.53)	0.87		
**BCR::ABL1**^(2)^ (negative/positive)	61/27	0.88 (0.60–1.81)	0.88		
**Sex category** (female/male)	50/43	1.17 (0.72–1.90)	0.52		
**Age at diagnosis**	93	1.05 (1.00–1.09)	**0.02**	1.05 (1.01–1.10)	**0.02**
**ECOG status**					
0–1	38	1	-		
2	41	1.29 (0.75–2.23)	0.36		
3	4	23.44 (6.62–83.05)	**<0.001**	18.49 (4.21–81.29)	**<0.001**
**CCI** (0–2/3–5) [21]	84/8	1.63 (0.70–3.81)	0.26		
**Therapy-related ALL** (no/yes)	81/11	0.44 (0.16–1.21)	0.11	0.48 (0.17–1.35)	0.17
**High-risk ALL**^(3)^ (no/yes)	25/65	0.91 (0.59–1.80)	0.91		
**Leukocytes at diagnosis**	86	1.000 (0.99–1.01)	0.63		
**Hemoglobin at diagnosis**	86	0.88 (0.75–1.04)	0.13	1.05 (0.88–1.25)	0.60
**Platelets at diagnosis**	86	0.997 (0.993–1.000)	0.06	0.996 (0.992–1.000)	0.06
**LDH at diagnosis**	85	1.000 (1.000–1.000)	0.39	-	-
**Bone marrow blasts at diagnosis**	74	1.00 (0.98–1.01)	0.96	-	-
**Peripheral blasts at diagnosis**	73	0.996 (0.988–1.003)	0.27	-	-
**Karyotype** (normal/abnormal)	22/58	1.33 (0.73–2.41)	0.35	-	-
**CNS infiltration** (no/yes)	76/6	1.25 (0.45–3.49)	0.67	-	-
**Extramedullary ALL at diagnosis** (no/yes)	79/13	0.82 (0.39–1.72)	0.59	-	-

Bold print indicates significant *p*-values. ^(1)^ Metric variables were utilized as continuous parameters. ^(2)^ Refers only to B-precursor ALL patients. ^(3)^ ≥1 of the following disease characteristics: leukocytosis > 30.000/µL at diagnosis, t (9;22) or t (4;11), pro-B-ALL, MRD positivity after 1st consolidation, late CR (>3 weeks after induction chemotherapy).

**Table 4:** Transplant characteristics of ALL patients > 55 years with B-precursor or T-ALL receiving allogeneic HSCT (33 pts).

Transplant Characteristics	Patients
**ALL subtype**, *n* ^(1)^ (%):	
pro-B-ALL	5/33 (15.2%)
c-ALL	25/33 (75.8%)
pre-B-ALL	2/33 (6.0%)
pro-T-ALL	1/33 (3.0%)
**Karyotype**, *n*/pts tested (%):	
normal	8/30 (26.7%)
1 or 2 aberrations	14/30 (46.6%)
complex (≥3 aberrations)	8/30 (26.7%)
**BCR::ABL1 at initial diagnosis**, *n*/pts tested (%):	
positive	17/33 (51.5%)
**Age at allogeneic HSCT** (years), median (range):	61.7 (55.6–67.6)
**Remission status at allogeneic HSCT**, *n* (%):	
1st CR	23/33 (70.0%)
2nd CR	7/33 (21.2%)
primary refractory	2/33 (6.1%)
refractory relapse	1/33 (3.0%)
**MRD status at allogeneic HSCT**^(2)^, *n*/pts tested (%):	
positive	12/26 (46.2%)
**HCT-CI**, *n*/pts tested (%):	
0–1	7/33 (57.6%)
2–3	8/33 (24.2%)
≥4	6/33 (18.2%)
**Conditioning regimen**, *n* (%):	
fludarabine/TBI (8 Gy)	31/33 (93.9%)
Other ^(3)^	2/33 (6.1%)
Donor type, *n* (%):	
MUD	16/33 (48.5%)
MRD	14/33 (42.4%)
MMUD	3/33 (9.1%)
**Stem cell source**, *n* (%):	
PBSC	31/33 (93.9%)
bone marrow	2/33 (6.1%)

ATG = anti-thymocyte globuline, Gy = Gray, GvHD = graft-versus-host disease, MUD = matched unrelated donor, MRD = minimal residual disease or matched related door, HCT-CI = hematopoietic cell transplantation comorbidity index, MMUD = mismatched unrelated donor, PBSC = peripheral blood stem cells. ^(1)^ General remark: “*n*” indicates the number of patients with data available for the respective category. ^(2)^ Concerning the 30 patients in CR. ^(3)^ Fludarabine/busulfane (1 patient), cyclophosphamide/treosulfane/etoposide (1 patient).

## Data Availability

The data generated in this study are available upon request from the corresponding author.
